# Scavengers of Nature

**Published:** 1863-07

**Authors:** 


"SCAVENGERS OF NATURE."—In the Report of the Board of Health of the City of Sacramento, California, for the year ending March 31, 1863, Dr. H. W. HARKNESS has the following remarks in respect to the influence of the terrible inundations of the winter of 1861–2 on the public health. They shew a remarkable provision in nature to counteract the evil effects which might otherwise arise from decaying vegetable matter:

"The sanitary condition of our city during the past year fully confirms me in the belief that the simple overflow of lands, although existing for months during the cold season, is not necessarily injurious to the health of the locality, providing there be not a large quantity of decomposing vegetable matter to poison the air upon its subsidence. Of the truthfulness of this theory, we have, as I think, ample evidence in the results of the flood we have of late encountered.

The inundation of December, 1861, took place at a time when our city was best prepared for it. The vegetation, already completely dessi[illegible]ated by the autumnal sun, had almost entirely disappeared; the deposit of alluvion or sedimentary matter buried the remainder. This sedimentary deposit, so pernicious to many localities, was really to us benificent. Had the supply of water been derived from the drainage of table lands, covered with lakes and swamps, bringing with it the debris of the previous summer's luxuriant growth, together with the miriad germs of plants of a microscopic and larger growth, the results would, no doubt, have been far different. But such was not the case. The water was derived exclusively from the American river, supplied from an exceedingly mountainous and sterile tract of country, furnishing but little of that material which would contaminate the atmosphere, and, besides the cottonwood and willow, but few of the germs for future growth.

"Owing to the above named causes and the low temperature of the waters, no deliterious effects were apparent on its subsidence. But there were those who believed that much mischief would result from the great number of pools left in every part of the city, and that when these became heated by a semi-tropical sun, fevers would prevail to an alarming extent. These sad forebodings were, happily, not realized.

"To the student of natural history, these small bodies of water were a great source of instruction and delight. In no place on this continent, perhaps, was there ever a better field for the observation of the microscopic world. In none, owing to favorable accidents, was there a greater diversity of the living organisms which the microseope reveals.

"These bodies of water were the favorite localities of the vast family of microscopic plants, whose germs are in many instances conveyed in the air. Amongst these may be mentioned the *Valvox Globator*, the *Bacillaria*, the *Diatomea Vulgare*, and the great family of the *Conjugatœa*, all of which are so minute as to be invisible to the unaided eye. These, if allowed to multiply without hindrance, would soon fill the water, and, although not noxious in their living and growing condition, would, on their death and decay, poison the air we breathe. Such results are counteracted by a new set of living organisms more wonderful still.

"All our citizens have noticed the green scum found floating on the surface of shallow ponds, from which they are accustomed to turn with disgust, and ascribe to it some injurious property. Push aside with a cane this apparently filthy covering, and you will observe the water, which but a day or two since was turbid, is now sweet and clear.

"Bring a portion of this green scum under the powers of the microscope, and it is found to consist of innumerable slender cylindrical-formed animalcules (the *Enchelia* of Ehrenberg), whose interiors impart the color from their distention with vegetable matter. And thus has this wonderful change been brought about by the innate wants of this living and growing spec, varying in size from 1-1200 to 1-400 of an inch; the matter which would otherwise decay and putrify is removed and its noxious effects on man and beast prevented. Other pools are alive with *Monads*, still more minute, whose bodies, from the same cause, impart to the water a tint of paler green. Others, again, are teeming with the *Rotifer Vulgaris* and the *Parameceum*, presenting the appearance of a greasy film as they float upon the surface of the water; the *Vorticella Convullaria*, with its wonderful apparatus for obtaining its food; the *Brachionis*, with its tortoise-like shell; the *Vibriona*, with elongated bodies so small as to require an instrument of great power to observe them, and many other varieties, all actively engaged either in the destruction of vegetable matter or in preying upon one another.

"In this manner the equilibrium between the minute vegetable and animal kingdom is preserved by the action of these living atoms, who have well earned the title of 'Scavengers of Nature.' The water is rendered sweet and clear, and many of the fairest portions of earth are made fit for the habitation of man."

